# Development of an Artificial Intelligence–Driven Three‐Dimensional Reconstruction Model of Renal Vasculature for Surgical Planning in Robot‐Assisted Partial Nephrectomy

**DOI:** 10.1002/rcs.70207

**Published:** 2026-07-10

**Authors:** Davide Brusa, Riccardo Bertolo, Francesco Ditonno, Greta Pettenuzzo, Lorenzo Pierangelo Treccani, Alessandro Veccia, Filippo Caudana, Francesco Fontana, Maria Angela Cerruto, Alessandro Antonelli

**Affiliations:** ^1^ University of Verona Urology Unit AOUI Verona Verona Italy; ^2^ Urology Unit Santissima Trinità Hospital Borgomanero Novara Italy

**Keywords:** 3D imaging, contrast‐enhanced CT, convolutional neural networks, digital reconstruction, nephron‐sparing surgery, renal vessel segmentation

## Abstract

**Background:**

This single‐centre study aimed to implement and internally validate a deep learning pipeline based on a 3D SegResNet architecture for automatic segmentation of renal vascular structures from contrast‐enhanced CT images, enabling accurate three‐dimensional reconstructions to support surgical planning in robot‐assisted partial nephrectomy.

**Methods:**

Ninety‐seven CT scans from patients with renal masses treated between January 2019 and September 2025 were included, with 68 used for training and 29 held out as an independent test set, never seen by the model during training. Imaging data included arterial and portal phases, and manual segmentations served as the ground truth. A 3D SegResNet architecture was implemented with spatial normalisation, isotropic resampling, data augmentation and tailored pre‐ and post‐processing.

**Results:**

The model achieved mean DICE scores of 0.86 for renal arteries and 0.81 for renal veins, with HD95 of 6.4 ± 2.8 mm and ASSD of 0.58 ± 0.22 mm for arteries, and HD95 of 8.7 ± 3.5 mm and ASSD of 0.94 ± 0.41 mm for veins, demonstrating high concordance with manual segmentation and correctly identifying most vascular structures. Performance was lower for distal intraparenchymal branches and in cases with multiple renal arteries or veins.

**Conclusions:**

Overall, this feasibility study supports the technical viability of automated, standardized and cost‐effective tools for surgical planning.

## 1 Introduction

The use of three‐dimensional (3D) models is becoming increasingly established in robotic kidney surgery, particularly in nephron‐sparing procedures [1, 2]. Their adoption has facilitated techniques such as superselective renal vessel clamping, which reduce intraoperative parenchymal injury and improve postoperative functional outcomes in patients undergoing partial nephrectomy [1, 2, 3, 4]. Despite their demonstrated clinical value in both preoperative planning and intraoperative guidance, 3D models are not yet widely available. This limited accessibility is largely due to the fact that, notwithstanding recent advances in artificial intelligence, these models cannot yet be generated fully automatically from computed tomography (CT) DICOM images. Current workflows still require substantial human intervention, especially during renal tissue segmentation and refinement of anatomical details critical for surgery [5]. This manual component significantly increases production costs and restricts 3D modelling availability to a limited number of centres with dedicated resources.

An active area of research is therefore focused on developing convolutional neural network–based artificial intelligence models capable of fully automatic segmentation and identification of anatomical structures on CT images [6, 7]. Deep learning approaches have shown increasing accuracy in recognizing both pathological and normal renal structures, offering a promising pathway toward fully automated 3D model generation. However, renal arterial and venous structures remain the most challenging targets for automated recognition. This difficulty stems from several factors, including the small caliber of distal vessels, complex three‐dimensional branching patterns, similarity in Hounsfield units between vascular structures and surrounding parenchyma, and significant inter‐patient anatomical variability [8, 9].

To date, the literature on automatic renal vessel segmentation remains limited. Although some studies have reported DICE similarity coefficients exceeding 80%, these results fall short of the approximately 90% threshold generally considered necessary for reliable clinical implementation [10, 11, 12]. The DICE coefficient quantifies the degree of overlap between automated and manually generated segmentations. In this context, our study integrates advanced pre‐processing techniques, extensively labeled CT datasets, and refined post‐processing algorithms to train an artificial intelligence model capable of accurately identifying renal vascular anatomy. Our objective is to achieve DICE performance comparable to or exceeding existing reports, thereby advancing the development of accurate, cost‐effective 3D models suitable for routine preoperative and intraoperative surgical planning [13, 14, 15].

## 2 Methods

### 2.1 Dataset

A comprehensive dataset of 97 contrast‐enhanced abdominal CT scans was collected from patients with renal masses who underwent surgical treatment at a single urologic unit between January 2019 and September 2025. The study was conducted in accordance with institutional and national ethical standards. The local ethics committee did not consider the application of the present aggregate of anonymous data from the institutional, routinely stored records. All DICOM images were systematically anonymised to ensure full patient confidentiality. The dataset was split a priori into a training set of 68 CT scans (70%) and a held‐out, independent test set of 29 CT scans (30%). The 29 test cases were never seen by the model during training; best‐epoch selection was based on training‐loss convergence on the 68‐case training cohort, and no metric computed on the 29 held‐out cases was used to inform model selection. The 29 test cases were therefore used exclusively for the final performance evaluation reported in this manuscript [14]. The cohort size is comparable to the most relevant public benchmark for this task (the KiPA22 challenge, ∼100 cases with annotated renal vasculature), reflecting the well‐documented scarcity of large, annotated CT datasets for renal vessel segmentation.

The cohort included a range of renal vascular anatomical configurations relevant for model development [16], comprising 47 cases with one renal artery and one renal vein, 15 cases with two renal arteries and one renal vein, four cases with one renal artery and two renal veins, one case with four renal arteries and one renal vein, and one case with two renal arteries and three renal veins (Table 1).

**TABLE 1:** Distribution of renal vascular anatomical configurations in the training and validation datasets.

Renal arteries	Renal veins	Cases (%)
Training set (*N* = 68)		
1	1	47 (69.1%)
2	1	15 (22.1%)
1	2	4 (5.9%)
4	1	1 (1.5%)
2	3	1 (1.5%)
Validation set (*N* = 29)		
1	1	16 (55.2%)
2	1	12 (41.4%)
1	2	1 (3.4%)

*Note:* Values are presented as number of cases and percentage within each dataset.

**FIGURE 1:**
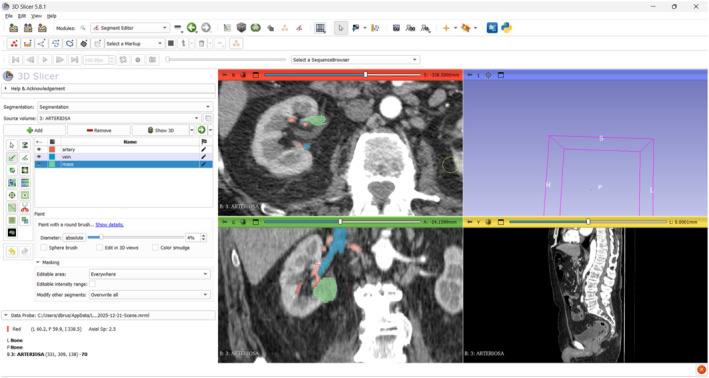
Manual ground‐truth segmentation performed in 3D Slicer (v5.8.1). Axial and coronal CT slices show expert annotation of renal artery (red), renal vein (blue), and renal mass (green). Only vascular segmentations were included in the training dataset; tumour delineation was generated for the anatomical context and excluded from model development.

All CT scans were acquired using GE Medical Systems scanners with a 512 × 512 acquisition matrix and a mean slice thickness of 2.5 mm. For each patient, two contrast‐enhanced phases were obtained: an arterial phase to visualise the renal arteries and a portal venous phase to delineate the venous system. Manual segmentation of the renal arteries and veins was performed by an experienced urologist using 3D Slicer software (v5.8.1; Figure 1). These manual annotations served as the ground truth for model training and validation [17].

Data preprocessing, convolutional neural network (CNN) training, and post‐processing were performed by an expert in deep learning and artificial intelligence engineering.

### 2.2 Preprocessing Pipeline

Images from both contrast‐enhanced phases were uploaded while preserving the original DICOM metadata. A standardized reorientation to the LPS (Left–Posterior–Superior) coordinate system was applied to ensure geometric consistency across scans. All images and corresponding segmentation masks were resampled to isotropic voxel spacing of 0.8 × 0.8 × 0.8 mm, using bilinear interpolation for image data and nearest‐neighbour interpolation for segmentation masks [18]. Hounsfield Unit (HU) values were clipped to the range [−1000, +1000] and normalised to [0, 1] to minimise radiologic variability across scanners and acquisition protocols [19].

The arterial and portal venous phases were merged into a multichannel volumetric input, generating a two‐channel tensor. This multiparametric fusion approach enables the model to learn complementary vascular information from both phases simultaneously: the arterial phase enhances arterial visualisation, whereas the portal venous phase improves venous delineation [20].

Extensive data augmentation was applied, including random scaling, axis‐aligned flipping, rotations between −20° and +20°, random contrast adjustments, intensity histogram modifications, automatic padding, and targeted patch extraction of 96 × 96 × 96 voxels containing renal vascular structures. Targeted sampling was particularly important, as vascular structures represent < 1% of total kidney volume [21, 22].

### 2.3 Model Architecture and Training

**FIGURE 2:**
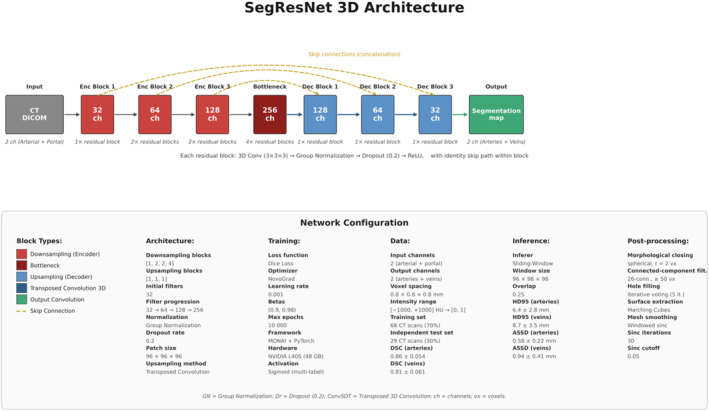
Complete segmentation framework and 3D SegResNet architecture. The pipeline comprises manual ground‐truth segmentation, pre‐processing, supervised training of a 3D SegResNet model, and post‐processing for volumetric reconstruction. The encoder–decoder architecture incorporates residual blocks, group normalisation, skip connections, and transposed convolutions. Dual‐channel CT input (arterial and portal phases) generates multi‐label outputs for renal arteries and veins. Final volumetric refinement and mesh extraction produce the reconstructed 3D vascular model displayed in a 3D slicer.

Renal vascular segmentation was performed using a three‐dimensional SegResNet architecture specifically designed to model complex, branching anatomical structures (Figure 2) [23]. The network combines residual learning with an encoder–decoder framework to capture multi‐scale contextual features. The architecture included two input channels (arterial and portal venous phases) and two output channels (arteries and veins). It was initialised with 32 convolutional filters and incorporated transposed convolutions for upsampling, Group Normalisation, and a dropout rate of 0.2. The network configuration comprised downsampling blocks (1, 2, 2, 4) and upsampling blocks (1, 1, 1). All layers employed volumetric (3D) convolutions, enabling fully three‐dimensional processing of the input data [24].

Model optimization was performed using Dice loss as the primary objective function, with background class exclusion, automatic one‐hot encoding, sigmoid activation for multi‐label segmentation, and a smoothing factor of 1 × 10^−5^. The NovoGrad optimiser was applied with a learning rate of 0.001 and beta parameters of 0.9 and 0.98 [25]. Training was conducted within the MONAI framework for up to 10,000 epochs, with best‐epoch selection based on training‐loss convergence within the 68‐case training cohort. The 29 independent test cases were never used to inform model selection.

### 2.4 Inference and Post‐Processing

Inference was performed using a SlidingWindowInferer with 96^3^ voxel windows and 25% overlap. Post‐processing included sigmoid activation, binarisation of probability maps, transformation back to native image space, and separation of arterial and venous channels.

The resulting probabilistic masks underwent a multi‐stage post‐processing pipeline to generate continuous and anatomically plausible vascular trees. Volumetric regularisation steps included three‐dimensional morphological closing to bridge 1–2 voxel discontinuities, connected‐component filtering to remove isolated fragments, and an iterative voting algorithm to fill internal cavities [26]. In detail: 3D morphological closing was applied independently on the arterial and venous probability channels using a small spherical structuring element of radius 2 voxels (approximately 1.6 mm at the isotropic resolution of 0.8 mm^3^), sized to bridge interruptions of one to two voxels along the vascular path that may arise in low‐contrast regions of the venous tree. Connected‐component filtering was then performed with 26‐connectivity, retaining only the components with volume above a minimum threshold of 50 voxels (approximately 25.6 mm^3^), so as to suppress isolated false‐positive fragments while preserving small accessory branches. Volumetric hole filling was carried out through an iterative 3D voting algorithm with 5 iterations, which closes internal cavities up to approximately 20 voxels (≈ 10.2 mm^3^) in size, a step essential for topologically correct segmentations. These parameters fall within the ranges typically reported in the literature for abdominal vascular post‐processing and were kept fixed across all test cases to avoid case‐specific tuning.

Surface reconstruction was performed using the Marching Cubes algorithm, followed by automatic mesh hole closure and windowed sinc smoothing to reduce surface irregularities while preserving vascular geometry. The windowed sinc filter was applied with 30 iterations and a pass‐band cutoff frequency of 0.05 to remove local voxelisation artefacts and produce a continuous surface without altering the true vascular morphology; these settings are consistent with the standard ranges reported in the literature for mesh smoothing of fine tubular anatomical structures.

### 2.5 Evaluation

Model performance was assessed using the Dice Similarity Coefficient (DSC), which ranges from 0 (no overlap) to 1 (perfect overlap) and quantifies the spatial agreement between predicted segmentations and reference (ground‐truth) annotations. To complement DSC, which is known to be insufficient on its own for thin tubular structures, two surface‐based metrics were also computed on the independent test set: the 95th‐percentile Hausdorff Distance (HD95), which captures large local errors while remaining robust to isolated outliers, and the Average Symmetric Surface Distance (ASSD), which quantifies the mean geometric deviation between the predicted and reference surfaces. Both metrics were computed channel‐wise (arteries and veins separately) in native image space, using the MONAI implementation, and are reported in millimetres.

All experiments were conducted using the MONAI framework and PyTorch library on NVIDIA L40S GPU equipped with 48 GB of memory. The computational environment was based on Python 3.9+ with CUDA 11.8 support.

## 3 Results

The trained convolutional neural network achieved the following DSC scores on the independent test dataset: 0.86 ± 0.054 for renal arteries and 0.81 ± 0.061 for renal veins (mean ± standard deviation). These numbers indicate that automatic three‐dimensional segmentation successfully predicts 86% of arterial vessel volume and 81% of venous vessel volume compared to expert manual segmentation. Surface‐based metrics on the same independent test set were as follows: HD95 = 6.4 ± 2.8 mm and ASSD = 0.58 ± 0.22 mm for renal arteries and HD95 = 8.7 ± 3.5 mm and ASSD = 0.94 ± 0.41 mm for renal veins. Surface metrics were consistently less favourable for veins than for arteries, mirroring the lower DSC and reflecting the lower contrast and greater anatomical variability of the venous tree in the portal phase.

**FIGURE 3:**
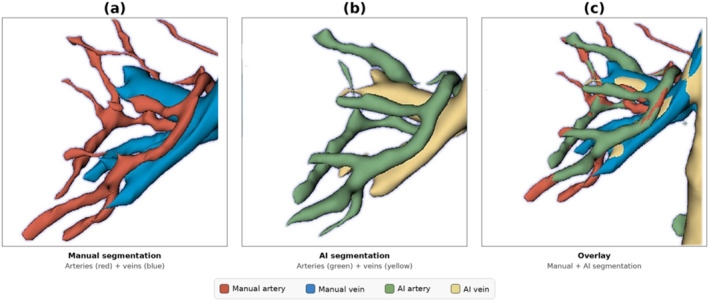
Representative case from the validation dataset: Three‐dimensional reconstruction visualised in 3D slicer. The left panel shows manual ground‐truth segmentation of renal arteries (red) and veins (blue). The middle panel displays the corresponding segmentation generated by the trained convolutional neural network (arteries in green, veins in yellow). The right panel shows the overlay of manual and AI‐based segmentations, illustrating spatial agreement between the reference and predicted vascular structures.

Visual assessment demonstrated accurate segmentation of the main renal vessels and primary branches. Reduced performance was observed in distal and smaller calibre segmental branches, particularly in regions where vessels penetrated deeply into the renal parenchyma and contrast enhancement was less distinct from surrounding tissue (Figure 3).

For renal arteries, the DSC variability (± 0.054) was observed across cases with different anatomical variants. Cases with multiple renal arteries showed greater segmentation variability than those with single‐artery configurations. To quantify this observation, we performed a post hoc subgroup analysis on the 29‐case test set, stratifying cases according to vascular complexity: standard anatomy (a single renal artery and a single renal vein; *n* = 16) and complex anatomy (≥ 2 renal arteries and/or ≥ 2 renal veins; *n* = 13). In the standard subgroup, mean DSC was 0.88 ± 0.04 for arteries and 0.82 ± 0.05 for veins; in the complex subgroup, mean DSC was 0.83 ± 0.07 for arteries and 0.79 ± 0.07 for veins. The corresponding surface metrics followed the same trend (arteries: HD95 5.6 ± 2.3 vs. 7.4 ± 3.1 mm; veins: HD95 8.0 ± 3.0 vs. 9.6 ± 3.9 mm). These differences should be interpreted with caution given the limited number of complex cases (*n* = 13) and the wide resulting confidence intervals, but they support the qualitative observation that multi‐vessel anatomies, including small‐calibre accessory arteries, remain the most challenging configurations for the current model. For renal veins, the DSC variability (± 0.061) was observed across cases with varying venous anatomy and contrast distribution.

## 4 Discussion

The achieved Dice Similarity Coefficients (DSCs) of 0.86 for renal arteries and 0.81 for renal veins demonstrate competitive performance in automatic renal vascular segmentation. Compared with previously published data, He et al. [11] reported DSC values of 0.89 ± 0.051 for arteries and 0.81 ± 0.072 for veins, the highest reported values to date (Table S1). Our arterial performance remains highly competitive, while venous segmentation matches published results with slightly lower variability, suggesting consistent performance across heterogeneous anatomical configurations. The HD95 and ASSD values reported in this study are broadly consistent, in order of magnitude, with those reported by the KiPA22 challenge participants for the same anatomical structures, when accounting for the difference between percentile‐based and maximum Hausdorff Distance. We frame our contribution as the clinical implementation and validation of a 3D SegResNet‐based pipeline within the MONAI framework, rather than a novel deep‐learning architecture; the methodological emphasis is therefore on the curation of an anatomically heterogeneous single‐centre cohort, the multi‐stage post‐processing pipeline producing surgically usable 3D meshes, and the feasibility assessment in the specific context of robot‐assisted partial nephrectomy.

Segmentation of renal vascular structures remains intrinsically challenging due to the small volumetric proportion of vessels relative to total renal parenchyma, the complex three‐dimensional branching architecture, and inter‐patient anatomical variability. Reduced performance in distal segmental branches and deeper parenchymal regions likely reflects decreased contrast differentiation and Hounsfield Unit values that become increasingly similar between vessels and surrounding cortical tissue. These intrinsic imaging characteristics remain a principal technical barrier in vascular segmentation tasks.

The inclusion of 97 contrast‐enhanced CT scans encompassing diverse vascular anatomies is an important methodological strength. Exposure to heterogeneous anatomical variants during training supports improved generalisability, as neural networks are inherently limited in recognising patterns not represented in the training dataset [27]. The 70%–30% training‐testing split aligns with established standards in medical image segmentation research [28], ensuring an appropriate balance between model learning and independent validation.

The preprocessing strategy contributed substantially to model stability and performance. Spatial standardisation to the LPS coordinate system, isotropic voxel resampling (0.8 × 0.8 × 0.8 mm), and intensity normalisation minimised geometric and radiologic variability across scans. The fusion of arterial and portal venous phases into a two‐channel volumetric input enabled the model to exploit complementary vascular information, improving discrimination between arterial and venous structures [20]. Additionally, targeted sampling of 96 × 96 × 96 voxel patches mitigated severe class imbalance, allowing the network to focus on learning about anatomically relevant vascular regions [21, 22].

The SegResNet architecture proved particularly suitable for this application. Three‐dimensional convolutions preserved spatial continuity in branching tubular structures, while the encoder–decoder framework maintained multiscale contextual information. Group Normalisation supported stable optimization with small batch sizes required by volumetric data, and a dropout rate of 0.2 reduced overfitting [29, 30]. The NovoGrad optimiser further contributed to stable convergence in deep 3D networks [25].

Post‐processing was essential to translate probabilistic outputs into clinically usable reconstructions. Volumetric morphological operations, connected‐component filtering, hole‐filling algorithms, and mesh refinement ensured topological continuity and anatomical plausibility. Surface extraction using the Marching Cubes algorithm followed by mesh regularisation generated smooth, continuous vascular trees suitable for three‐dimensional visualisation and potential surgical planning applications [26].

Beyond numerical performance, automated renal vascular recognition enables fully automated three‐dimensional reconstruction with minimal human intervention. This represents a meaningful step towards standardized, reproducible, and cost‐effective 3D surgical planning tools. Current manual or semi‐manual segmentation workflows are labour‐intensive and limit access to advanced 3D modelling to highly specialised centres. However, the present study should be interpreted as demonstrating the technical feasibility of automated renal vessel segmentation and 3D reconstruction rather than its direct clinical utility. Although the achieved segmentation accuracy is encouraging, it remains unclear whether this level of performance is sufficient to support clinical decision‐making or to improve surgical outcomes in robot‐assisted partial nephrectomy.

A validated automated approach may facilitate broader dissemination of 3D planning in nephron‐sparing surgery, particularly in robot‐assisted partial nephrectomy, where precise understanding of segmental vascular anatomy is critical for superselective clamping and parenchymal preservation [31, 32].

Several limitations must be acknowledged. The dataset remains modest in size for deep learning applications, and all scans were acquired at a single centre using GE Medical Systems scanners with standardized acquisition parameters. This sample size, however, is comparable to that of the largest publicly available benchmark for the same task (the KiPA22 challenge), reflecting the well‐documented scarcity of annotated CT datasets for renal vasculature. External validation of heterogeneous scanners and multicenter datasets is necessary to confirm generalisability. The retrospective design limits the assessment of real‐world clinical deployment. Ground‐truth annotations were generated by a single experienced urologist using 3D Slicer (v5.8.1) following a standardized internal protocol. While this approach ensured internal consistency, it precluded any quantification of inter‐observer variability, which is known to influence both training and evaluation in vessel segmentation tasks; the reported metrics should therefore be interpreted relative to a single reference standard, and the residual annotation bias cannot be excluded. A multi‐annotator protocol with consensus or majority‐voting fusion involving radiology colleagues is planned for the next phase of the study, and is expected to provide a more robust ground‐truth reference and an estimate of human‐level variability for benchmarking. We also note that, for thin tubular structures, branch‐detection and centreline‐continuity metrics would complement the surface‐based metrics reported here; their integration is planned for a follow‐up study specifically focused on distal, intraparenchymal vessel reconstruction.

Future multicenter collaborations incorporating heterogeneous imaging protocols are warranted to enhance robustness and generalisability. Importantly, prospective studies evaluating the integration of these automated reconstructions into real surgical planning workflows are required to determine whether the observed technical performance translates into clinically meaningful benefits.

## 5 Conclusion

This feasibility study reports the implementation and independent‐test‐set validation of a 3D SegResNet‐based pipeline that achieved competitive performance in automatic segmentation of renal arterial and venous vessels from contrast‐enhanced CT scans. Volumetric (DSC) and surface‐based (HD95, ASSD) metrics, together with the subgroup analysis by vascular complexity, support the technical feasibility of automated three‐dimensional renal vascular reconstruction for surgical planning in robot‐assisted partial nephrectomy. Further multicenter validation, multi‐annotator ground‐truth protocols, and prospective clinical integration studies are warranted before routine implementation.

## Funding

The authors have nothing to report.

## Ethics Statement

The study was conducted in accordance with institutional and national ethical standards. The local ethics committee did not consider the application of the present aggregate of anonymous data from the institutional, routinely stored records.

## Consent

Informed consent was obtained from individual participants included in the study.

## Conflicts of Interest

The authors declare no conflicts of interest.

## Supporting information


**Table S1:** Reported dice similarity coefficient values for automatic renal vessel segmentation in previously published studies [9].

## Data Availability

The data supporting the findings of this study are available from the corresponding author upon reasonable request.
