# Supplementary material for: Development of an Artificial Intelligence–Driven Three‐Dimensional Reconstruction Model of Renal Vasculature for Surgical Planning in Robot‐Assisted Partial Nephrectomy
Source: Int J Med Robot. 2026 Jul 10;22(4):e70207. doi: 10.1002/rcs.70207 (PMC13354272; doi:10.1002/rcs.70207)
Supplement: Supplementary file 1 — Table S1: Reported dice similarity coefficient values for automatic renal vessel segmentation in previously published studies [9]. [file RCS-22-e70207-s001.docx]

| Method | Vein | Artery |
| --- | --- | --- |
|  | DSC (%) | DSC (%) |
| V-Net (Milletari et al., 2016) | 76.4 ±9.2 | 84.3 ± 4.5 |
| 3D U-Net (Cicek et al., 2016) | 73.7±13.5 | 80.2±8.2 |
| Res-U-Net (Li et al., 2018) | 76.8±7.9 | 84.5±8.6 |
| Kid-Net (Taha et al., 2018) | 75.4±10.4 | 78.0±7.1 |
| VFN (EN) (Xia et al., 2018) | 76.9±9.0 | 81.8±6.1 |
| DenseBiasNet (He et al., 2020) | 75.3±11.3 | 86.1±8.0 |
| Our experience | 81.0±6.1 | 86.0±5.4 |

**Supplementary Table 1.** Reported Dice Similarity Coefficient values for automatic renal vessel segmentation in previously published studies [9].
